# HS-173, a novel PI3K inhibitor suppresses EMT and metastasis in pancreatic cancer

**DOI:** 10.18632/oncotarget.12871

**Published:** 2016-10-25

**Authors:** Marufa Rumman, Kyung Hee Jung, Zhenghuan Fang, Hong Hua Yan, Mi Kwon Son, Soo Jung Kim, Juyoung Kim, Jung Hee Park, Joo Han Lim, Sungwoo Hong, Soon-Sun Hong

**Affiliations:** ^1^ Department of Biomedical Sciences, College of Medicine, Inha University, Sinheung-dong, Jung-gu, Incheon 400-712, Republic of Korea; ^2^ Department of Chemistry, Korea Advanced Institute of Science and Technology (KAIST), and Center for Catalytic Hydrocarbon Functionalizations, Institute of Basic Science (IBS), Daejeon 34141, South Korea

**Keywords:** pancreatic cancer, metastasis, EMT, PI3K, HS-173

## Abstract

Pancreatic cancer is one of the most aggressive solid malignancies prone to metastasis. Epithelial-mesenchymal transition (EMT) contributes to cancer invasiveness and drug resistance. In this study, we investigated whether HS-173, a novel PI3K inhibitor blocked the process of EMT in pancreatic cancer. HS-173 inhibited the growth of pancreatic cancer cells in a dose- and time-dependent manner. Moreover, it significantly suppressed the TGF-β-induced migration and invasion, as well as reversed TGF-β-induced mesenchymal cell morphology. Also, HS-173 reduced EMT by increasing epithelial markers and decreasing the mesenchymal markers by blocking the PI3K/AKT/mTOR and Smad2/3 signaling pathways in pancreatic cancer cells. In addition, HS-173 clearly suppressed tumor growth without drug toxicity in both xenograft and orthotopic mouse models. Furthermore, to explore the anti-metastatic effect of HS-173, we established pancreatic cancer metastatic mouse models and found that it significantly inhibited metastatic dissemination of the primary tumor to liver and lung. Taken together, our findings demonstrate that HS-173 can efficiently suppress EMT and metastasis by inhibiting PI3K/AKT/mTOR and Smad2/3 signaling pathways, suggesting it can be a potential candidate for the treatment of advanced stage pancreatic cancer.

## INTRODUCTION

Pancreatic cancer is one of the most aggressive solid malignancies with an extremely poor prognosis of an overall 5-year survival rate of <5% [1]. Without a specific diagnostic marker and symptom in early stage, pancreatic cancer is often diagnosed at an advanced/late stage and only palliative measures can be offered [2]. Because of the late onset of symptoms, only 10%–15% of patients are presented with resectable disease, whereas the remaining are present with either locally advanced unresectable or metastatic disease. Namely, more than 80% of pancreatic cancer patients are present with locoregional spread and/or distant metastasis, being the main cause of cancer-related death [3]. The role of chemotherapy in metastatic pancreatic cancer is to relieve symptoms and to improve survival [4]. Nevertheless, the lack of effective chemotherapies and drug resistance contributes to the high mortality of patients diagnosed with metastatic pancreatic cancer [5, 6]. Therefore, the development of more effective treatments and approaches for pancreatic cancer with metastasis is urgently needed.

The molecular mechanism involved in metastasis has been shown to dysregulate the signaling pathways that control epithelial-mesenchymal transition (EMT). The process of EMT has been proposed to allow immotile epithelial cells with cellular polarity to dissociate from each other and undergo a morphological transformation into cells with a fibroblast-like mesenchymal shape in order to engage in program of the cell motility, affecting cell migration [7]. EMT endows cells with migratory and invasive properties, inducing resistance to conventional chemotherapy [8, 9]. Especially, in pancreatic cancer, the expression of EMT markers such as Ncadherin, Vimentin, and transcription factors including ZEB1, Snail, and Twist have been increased in surgically resected pancreatic cancer sepecimens but not in the normal noncancerous pancreatic tissue [9-11]. Moreover, the presence of EMT in pancreatic cancer has been reported to be frequently associated with undifferentiated phenotype and overall poor survival compared with tumors without EMT [11, 12]. As mentioned above, EMT induces drug resistance in cancer cells, which gives rise to the acceleration of metastasis [6]. Thus, suppression of EMT is very crucial to improve drug response and to block metastasis in cancer therapies.

Transforming growth factor-β (TGF-β) is a potent inducer of EMT and positive regulator of tumor progression and metastasis [13, 14]. TGF-β has been shown to activate various downstream pathways including phosphatidylinositol-3-kinase (PI3K), Smads, and MAPK, which are involved in TGF-β-induced EMT [15-18]. In particular, activation of PI3K/AKT pathway has emerged as a central signaling pathway in regulating EMT [19].

PI3K signaling plays an important role in cell cycle, survival, metabolism, and motility [20]. Aberrations of the PI3K signaling induces the pathogenesis of numerous cancers by altering cell growth and apoptosis [1]. Vichalkovski *et al.* have reported that activation of AKT upregulated the expression of Twist, subsequently decreasing chemotherapy-induced DNA damage, and inhibiting apoptosis [21]. Moreover, phosphorylated AKT is positively correlated with the high level of Twist as well as low expression of Ecadherin in oral squamous cell carcinoma [22]. In addition, the treatment of AKT inhibitor decreased the expression of Snail and Twist [23, 24] and eventually promoting the expression of Ecadherin, reducing the expression of Vimentin; restored the polygonal shape of cells, and stimulated the mesenchymal-epithelial transition (MET).

Given that the PI3K/AKT signaling activates the process of EMT and tumor metastasis, we hypothesized that targeting the PI3K/AKT signaling pathway may suppress EMT. Therefore, we explored whether HS-173 [25-27], a novel PI3K inhibitor might inhibit TGF-β-induced EMT and metastasis *in vitro* and *in vivo*. Our study revealed that HS-173 could efficiently suppress TGF-β-induced migration, invasion, and metastasis by inhibiting EMT process through the regulation of PI3K/AKT/mTOR and Smad2/3 signaling pathways, suggesting that HS-173 is a potential candidate for advanced stage treatment of pancreatic cancer with metastasis.

## RESULTS

### HS-173 inhibits cancer cell growth and colony formation in pancreatic cancer cells

**Figure 1:**
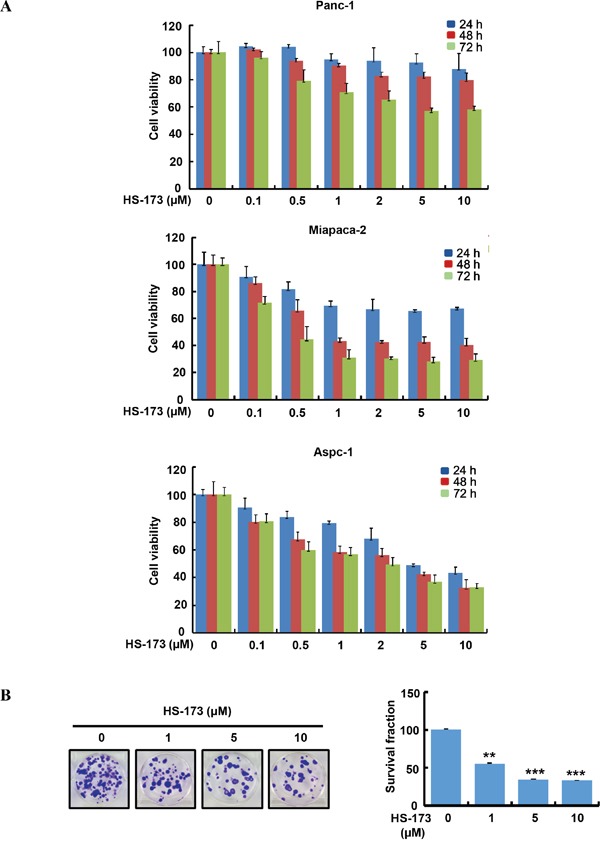
HS-173 suppressed the growth of pancreatic cancer cells **A.** HS-173 reduced the cell viability of Panc-1, Miapaca-2 and Aspc-1 cells. The cells were treated with HS-173 at the indicated concentration for 24 h, 48 h and 72 h before MTT assays were performed. Data from three independent experiments were represented as the mean ± S.D. **B.** HS-173 inhibited the colony formation dose-dependently in Miapaca-2 cells. After HS-173 treatment for 24 h, cells were processed for clonogenic survival assays at the end of experiment (14 days). Data are represented as the mean ± S.D. from triplicate experiments. **P<0.01 and ***P<0.001 vs control.

In order to evaluate the effect of HS-173 on the cell growth of pancreatic cancer, three cell lines (Panc-1, Miapaca-2 and Aspc-1) were used. When pancreatic cancer cells were treated with various concentrations (0.1-10 μM) of HS-173, it reduced the cell viability in a dose- and time-dependent manner (Figure 1A). In particular, 1 μM of HS-173 inhibited the cell growth by 40-50% in Miapaca-2 and Aspc-1 cells at 48 h. To further determine the sensitivity of HS-173, we performed clonogenic assay in Miapaca-2 cells for 14 days. In agreement with the MTT assay, HS-173 showed a significant drug response by the inhibition of colony formation in pancreatic cancer cells dose-dependently. Additionally, we observed that colony formation by HS-173 was less than 50% in Miapaca-2 cells at dose of 1 μM (Figure 1B).

### HS-173 inhibits TGF-β-induced cell migration and invasion in pancreatic cancer cells

**Figure 2:**
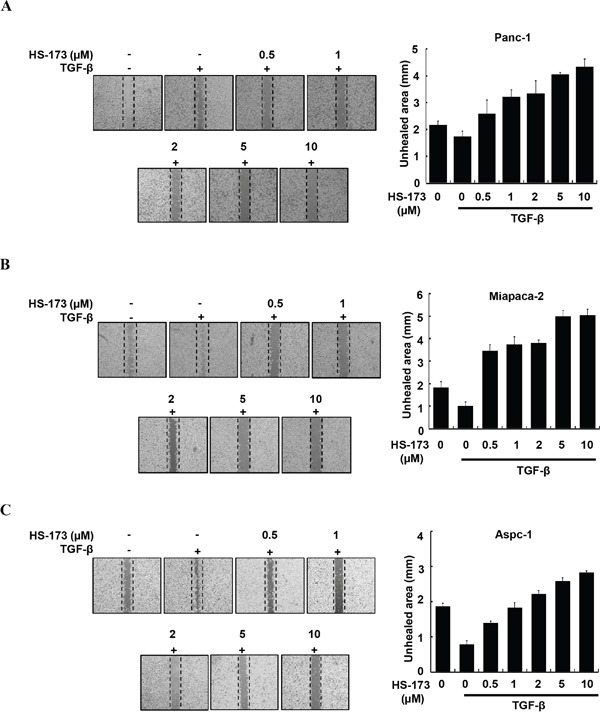
HS-173 inhibited TGF-β-induced migration of pancreatic cancer cells Representative images of wound healing assay of Panc-1 **(A),** Miapaca-2 **(B)** and Aspc-1 **(C)** cells after 16 h treatment with or without TGF-β (10 ng/mL) alone, or along with indicated dose of HS-173 for 24 h. For quantification, analysis of the unhealed area at the indicated dose of HS-173 was performed. All images were captured at 200x magnification. Data are represented as the mean ± S.D. from triplicate experiments.

**Figure 3:**
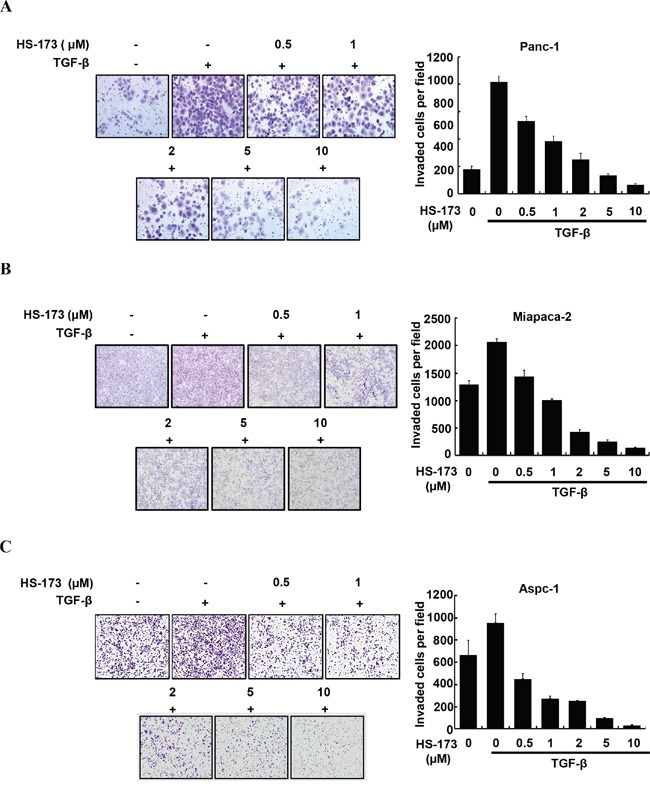
HS-173 reduced TGF-β-induced invasion of pancreatic cancer cells Representative images of invaded cells of Panc-1 **(A),** Miapaca-2 **(B)** and Aspc-1 **(C)** through matrigel coated boyden chamber after HS-173 treatment for 48 h are shown. The number of invaded cells were presented as the mean ± S.D. from triplicate experiments. Images were captured at 200x magnification.

As enhanced migration has been shown to occur during EMT [28], we performed wound healing assay to identify the effect of HS-173 on the migration of pancreatic cancer cells. For the significant induction of EMT *in vitro*, we used TGF-β (10 ng/mL). In three pancreatic cancer cell lines, TGF-β treated group was healed over 90% of the wound area within 16 h, whereas cells in control group were slowly migrated. However, HS-173 successfully suppressed TGF-β-induced cell migration dose-dependently in all pancreatic cancer cell lines (Figure 2). Next, as invasive ability is one of the major hallmarks of cancer cell, we identified the effect of HS-173 on the invasion of pancreatic cancer cells using transwell invasion assays. As shown in Figure 3, TGF-β induced the invasive capacity of cancer cells, which was inhibited by HS-173 co-treatment. Collectively, these results indicate that HS-173 effectively prevents TGF-β-induced cell miagration and invasion.

### HS-173 suppresses TGF-β-induced epithelial mesenchymal transition (EMT)

**Figure 4:**
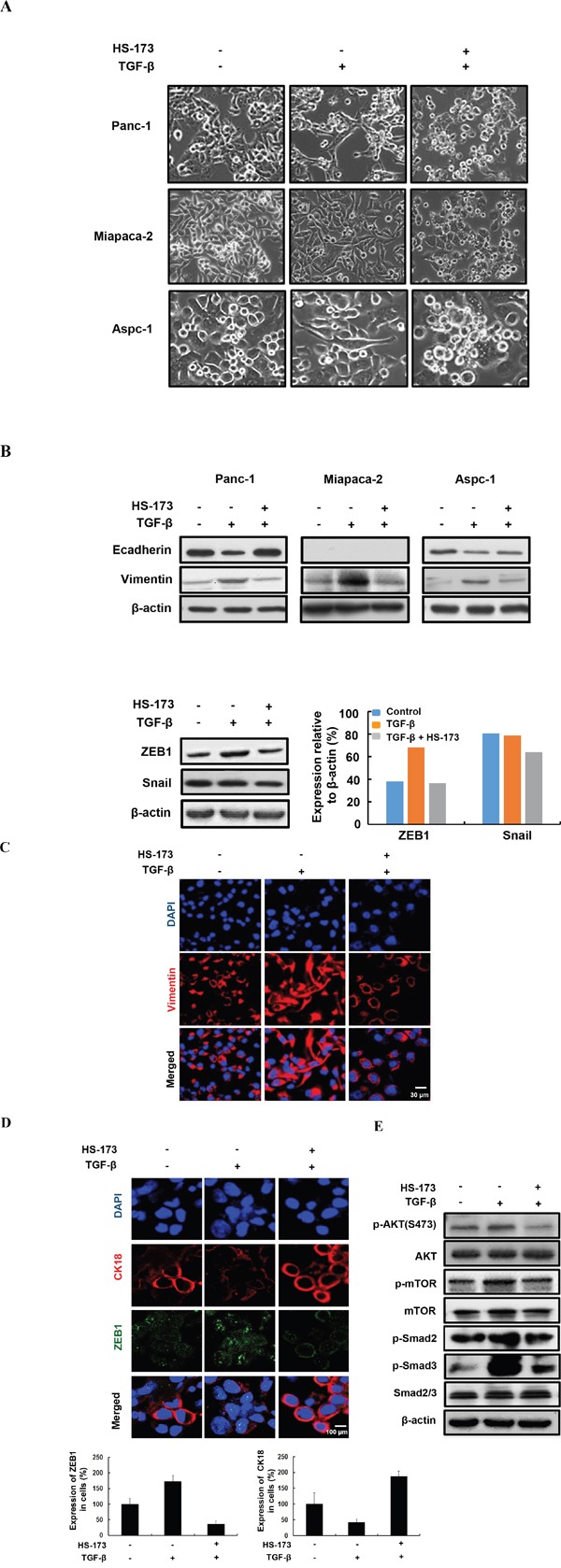
HS-173 inhibited TGF-β-induced EMT *in vitro* **A.** Morphology of indicated cells were shown under phase contrast microscopy. The cells were treated with or without TGF-β (10 ng/mL) alone, or along with HS-173 (5 μM) for 24 h. Images were captured at 400x magnification. **B.** The EMT-related proteins were detected by Western blot analysis in Panc-1, Miapaca-2, and Aspc-1 cell lines. EMT transcriptional factors including ZEB1 and Snail were detected by Western blot analysis in Panc-1 cells. **C** and **D.** The expression of EMT markers were evaluated in Panc-1 cells by immunofluorescence staining after HS-173 treatment for 6 h. **E.** The effect of HS-173 on TGF-β-induced protein expression of p-AKT, p-mTOR, p-Smad2, and p-Smad3 were investigated in Panc-1 cells by Western blot analyses after HS-173 treatment for 6 h.

TGF-β is a positive regulator of EMT in cancer, as indicated by a morphological change to spindle-shaped appearances, decreased expression of epithelial markers and increased expression of mesenchymal markers. Therefore, we investigated whether HS-173 could change EMT related morphology after EMT induction by TGF-β in pancreatic cancer cells. As shown in Figure 4A, the cells treated with TGF-β lost their typical epithelial morphology and their ability to grow in clusters and acquired a mesenchymal phenotype with a flat and spindle-like morphology. However, the cells treated with HS-173 were more cuboidal and tightly adherent in TGF-β-induced pancreatic cancer cells.

In epithelial cancer cells, the decreased Ecadherin and the increased Vimentin expression are known as the main EMT characteristics, which is responsible for the progression of EMT such as cell migration and metastasis [29]. To explore whether HS-173 affected the expressions of Ecadherin and Vimentin, we measured the expression using Western blotting. As a result, we found that HS-173 recovered the MET process as follows: the expression of Ecadherin was increased, whereas that of Vimentin was strongly decreased in TGF-β-induced pancreatic cancer cells. As mentioned above, ZEB1, Snail, and Twist repressed Ecadherin, and their enhanced expression is regarded as key events in the EMT process [30]. In our study, ZEB1 was highly activated by TGF-β, which was significantly inhibited by HS-173 (Figure 4B). This result was also confirmed by immunofluorescence data (Figure 4D). However, the expression of Snail showed a slight inhibition after HS-173 treatment in TGF-β-induced pancreatic cancer cells (Figure 4B). As shown in Figure 4C, HS-173 obviously inhibited the expression of Vimentin, showing spider web-like stress fibers, which is a typical characteristic of mesenchymal cells. The fact that HS-173 significantly inhibited EMT process raised the question how the effect may have been mediated. As a PI3K inhibitor, HS-173 can be predicted to interrupt the PI3K/AKT axis, and consequently block the activation of downstream effectors such as AKT and mTOR. Therefore, to investigate whether the anti-EMT effect of HS-173 was mediated by the inhibition of PI3K/AKT and TGF/Smads signaling pathways, we explored the phosphorylation status of PI3K/AKT and TGF/Smads signaling pathways, respectively. As expected, the treatment of HS-173 effectively inhibited p-AKT and p-mTOR, and subsequently decreased the p-Smad2 and p-Smad3, implying that HS-173 had anti-EMT effect by regulation of both PI3k/AKT and TGF/Smads signaling (Figure 4E).

### HS-173 prevents TGF-β-induced epithelial mesenchymal transition (EMT) in 3D spheroids

**Figure 5:**
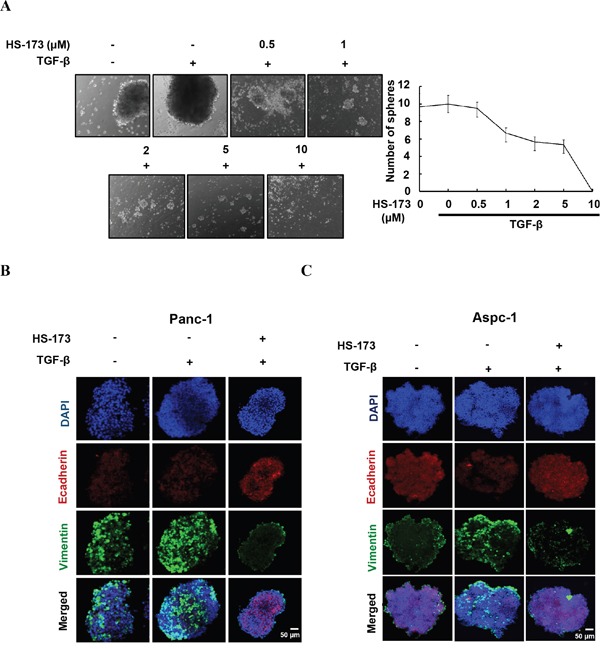
HS-173 reduced the growth of TGF-β-induced tumor spheroids **A.** Sphere formation assays were perfomed in Panc-1 cells. Tumor spheroids were treated with or without TGF-β (10 ng/mL) alone, or along with different doses of HS-173 (0.5-10 μM) for 24 h. **B** and **C.** 3D tumor spheroids using modified hanging drop method were formed and treated with or without TGF-β (10 ng/mL) alone, or along with HS-173 (5 μM) for 48 h. The expression of Ecadherin and Vimentin were observed in Panc-1 and Aspc-1 cells by immunofluorescence assay.

As conventional two-dimensional (2D) culture models lacks realistic complexity to reflect human tumor biology, we investigated the effect of HS-173 on three dimensional (3D) culture. In sphere formation assay, Panc-1 cells were treated with either TGF-β alone or along with different doses of HS-173 (Figure 5A). Our study showed that the spheres of TGF-β treated group became more compact than that of control group, whereas HS-173 along with TGF-β reduced the sphere size dose-dependently. To further confirm these results, we evaluated the expression of Ecadherin and Vimentin in 3D spheroids cultures using modified hanging drop method. After the exposure to 10 ng/mL of TGF-β, the expression of Vimentin was rapidly induced and HS-173 reversed this up-regulation, whereas Ecadherin was also highly expressed in HS-173-treated group (Figure 5B).

### HS-173 inhibits primary tumor growth and EMT *in vivo*

**Figure 6:**
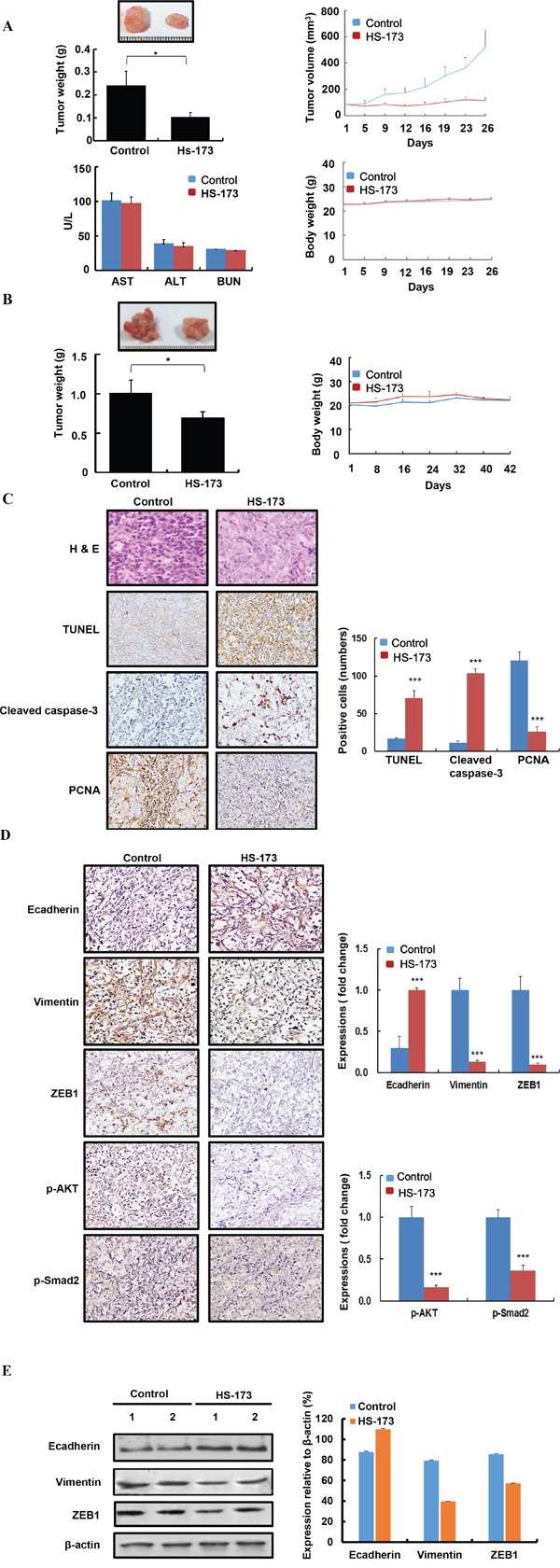
HS-173 inhibited tumor growth of pancreatic cancer and EMT *in vivo* **A.** Tumor volume/weight and body weight were measured every 2 days in Panc-1 pancreatic cancer xenograft mouse models. After 26 days of treatment, tumors were excised and blood was collected to evaluate the level of AST, ALT, and BUN. **B.** Orthotopic mice models were also established by the inoculation of 8×10^6^ Panc-1 cells into the pancreas. After 42 days of treatment, tumors were excised from pancreas. In two *in vivo* mouse models, BALB/c nude mice were divided into two groups (control and HS-173) and mice were treated with vehicle alone or along with HS-173 (10 mg/kg) intraperitoneally thrice a week. Data are represented as the mean ± S.D. **C** and **D.** *In vivo* effect of HS-173 on proliferation, apoptosis in a pancreatic xenograft mouse models. Isolated pancreatic tumors were sectioned at 8-μm thick and stained with PCNA, TUNEL, cleaved caspase-3, Ecadherin, Vimentin, ZEB1, p-AKT and p-Smad2 including H&E staining at captured at 200x magnification. For quantification, we analyzed an optical density of cell stained with PCNA, TUNEL, cleaved caspase-3, Ecadherin, Vimentin, ZEB1, p-AKT and p-Smad2. **E.** *In vivo* effect of HS-173 on EMT in the tumors isolated from pancreatic xenograft mouse models. Tumors were excised and processed for Western blotting. Data were represented as the mean ± S.D. *P<0.05 and ***P<0.001 vs control.

To further determine whether HS-173 is involved in tumorigenesis *in vivo*, xenograft and orthotopic pancreatic tumor models were used in the Balb/c nude mice. For establishing xenograft tumor, Panc-1 cells were inoculated subcutaneously in the right flank of the mice and in orthotopic model, the cells were inoculated into the pancreas. In xenograft and orthotopic models, mice were treated by HS-173 intraperitoneally at a dose of 10 mg/kg (n=8, each group) after 10 days of inoculation. In this study, we observed that HS-173 dramtically reduced tumor volume and weight, compared with the control group in two mouse models (Figure 6A and 6B). Moreover, any significant changes in the body weight or any adverse effects were not observed in animals treated with HS-173. Moreover, aspartate aminotransferase (AST), alanine aminotransferase (ALT), and blood urea nitrogen (BUN) values in serum were not significantly changed as well as there were no abnormality in the H&E of liver tissues (data not shown). Also, HS-173 significantly increased expression of TUNEL, cleaved caspase-3 along with decreased expression of PCNA in tumor tissues (Figure 6C). To further confirm whether or not HS-173 inhibits tumor growth through the regulation of EMT, we identified the expression levels of Ecadherin, Vimentin, ZEB1 along with p-AKT and p-Smad2. In agreement with our previous *in vitro* result, Ecadherin was increased, whereas Vimentin and ZEB1 were downregulated in the HS-173 treated group (Figure 6D and 6E). Furthermore, HS-173 treatment decreased p-AKT and p-Smad2 in tumor tissues. Taken together, our results demonstrate that HS-173 has potent anti-tumor efficacy by inhibiting EMT via regulation of PI3K/AKT and TGF/Smads pathways.

### HS-173 reduces metastasis in cancer metastatic mouse models

**Figure 7:**
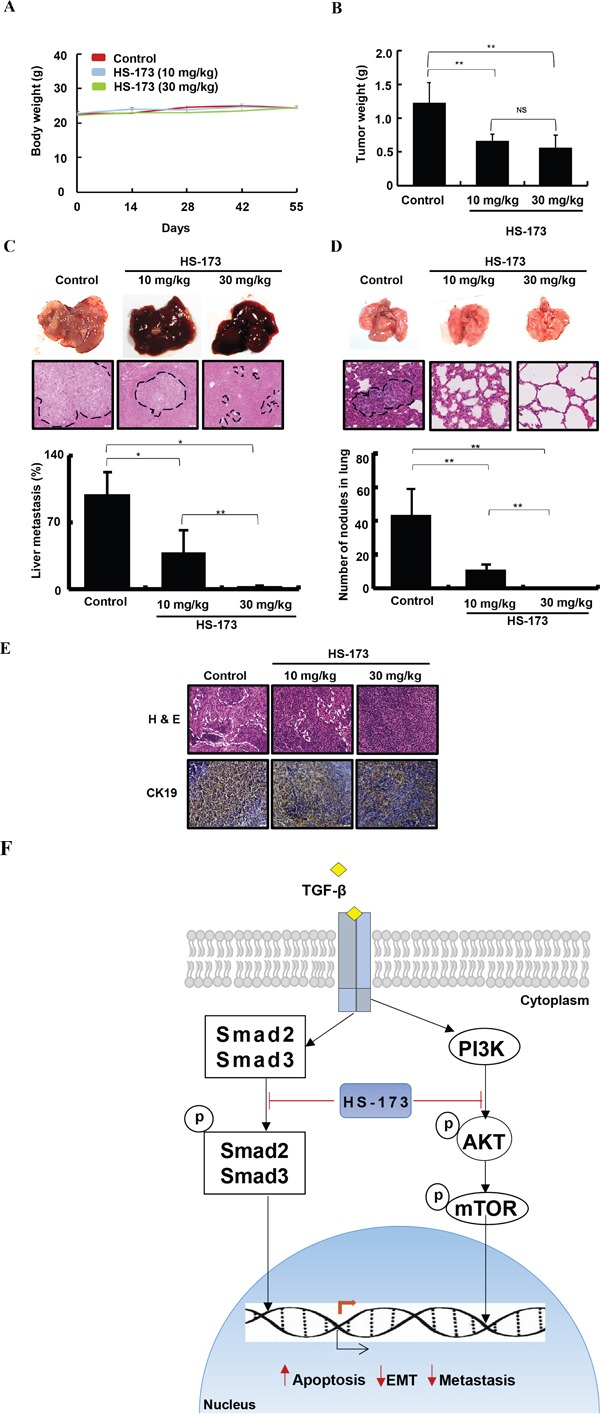
HS-173 suppressed metastasis in mouse cancer metastatic models Metastasis mouse models were established in BALB/c nude mice transplanting Miapaca-2 cells into the spleen. After 30 days of inoculation, HS-173 was treated at doses of 10 mg/kg and 30 mg/kg (n=7, each group) for 25 days. **A.** Body weight and **B.** primary tumor weight were measured in metastasis mouse models. **C.** Livers and **D.** lungs were excised and processed for H&E staining following to metastatic scoring. The arrows and dotted lines indicates metastatic nodules and metastatic area, respectively. **E.** CK19 expression was identified in lymph nodes of metastatic mouse models using immunohistochemistry. Images were captured at 200x magnification. Data are shown as the mean ± S.D. *P<0.05 and **P<0.01 vs control. **F.** In schematic presentation of the effect of HS-173 on EMT and metastasis, HS-173 inhibited the PI3K/AKT and Smad2/3 pathways and finally suppressed cancer cell growth, invasion and metastasis. The arrows and blunt ends indicate activation and inhibition, respectively.

Based on the *in vivo* findings, we extended our study to metastasis models, establishing a long term cancer metastatic models with Balb/c nude mice transplanting Miapaca-2 cells into the spleen. After 30 days of inoculation, HS-173 was treated at doses of 10 mg/kg and 30 mg/kg (n=7, per group) for 25 days. The liver, lung, and lymph node along with primary tumors were excised and the metastatic lesions were determined by H&E staining (Figure 7). Our results showed that the metastatic burdens on the lung and liver were significantly decreased by HS-173 treatment (10 and 30 mg/kg). As previous studies have shown that CK19 expression is correlated with lymph node metastasis in various solid malignancies [31-33], we identified its expression in the lymph node. As shown in Figure 7E, the CK19 expression was decreased by HS-173 treatment.

## DISCUSSION

The issues of tumor recurrence, drug resistance, enhanced invasion and metastasis remain a challenge in the treatment and clinical management of pancreatic cancer. Despite the efforts to better understand the metastasatic progression of pancreatic cancer, more effective treatments have been limited due to the difficulty in the identification of functionally relevant targets of the disease. EMT is thought to generate metastasis and contribute to therapy resistance. Therefore, the EMT pathway is of great therapeutic interest in the treatment of cancer and could be targeted to prevent tumor dissemination in patients with high risk of developing metastatic lesions. Recent studies have suggested that some novel cancer drugs may be capable of controlling the spread of carcinoma via EMT inhibition [34, 35]. Recently, PI3K has gained widespread attention as a potential target for preventing and treating metastatic tumors associated with EMT. Nevertheless, no PI3K/AKT inhibitors have been approved for treating pancreatic cancer patients with metastasis. Accordingly, we sought to analyze the effects of HS-173, a PI3K inhibitor on the EMT in pancreatic cancer and to identify its possible mechanism of action. Herein, we found that HS-173 inhibited TGF-β-induced migration and invasion, as well as EMT-related proteins by the suppression of PI3K/AKT/mTOR signaling pathway in parallel with the TGF-β/Smad2/3 signaling, leading to the inhibition of metastasis in pancreatic cacner *in vivo* models.

EMT can be triggered or regulated by various growth factors such as TGF-β, FGF, HGF, PDGF, Wnt, and Notch [36]. Among them, TGF-β has been identified as a primary driver, and transcription factors such as ZEB1 and Snail has been shown to be essential in invoking the broad changes of gene expressions associated with the mesenchymal cell type [37]. During tumorigenesis, TGF-β signaling has increased motility, invasiveness and ultimately metastasis. This event appears to be the consequence of EMT mediated by the PI3K/AKT signaling pathway [38]. TGF-β has stimulated AKT phosphorylation via PI3K, resulting in EMT characterized by reorganization of actin fibers (Vimentin and Fibronectin) and delocalization of Ecadherin [16]. Therefore, to understand the signaling mechanism mediated by HS-173, a PI3K inhibitor on EMT process, we first identified the expression of Vimentin and Ecadherin, representative EMT markers and then observed the changes of p-AKT and p-mTOR, which are downstreams of the PI3K/AKT signaling pathway in TGF-β-induced pancreatic cancer cells. Our study showed that TGF-β acivated the AKT/mTOR signaling and also increased the expression of Vimentin as well as decreased that of Ecadherin. However, HS-173 dramatically reversed these phenomena, resulting in the inhibition of mobility and invasiveness in pancreatic cancer cells. Other PI3K inhibitors, LY294002 and BEZ-235 have also inhibited EMT process by increasing expression of Ecadherin and decreasing those of Vimentin and Fibronectin in lens epithelial cells and non-small cell lung cancer cells [39, 40]. These results signify that the effect of HS-173 on EMT is closely related to its inhibitory activity on the PI3K/AKT/mTOR signaling. In fact, the activation of PI3K/AKT in cancer is responsible for the dysregulation of cell growth, survival, proliferation, and notably EMT. For instance, EMT driven by activated AKT involves the loss of cell-cell adhesion, morphological changes, loss of cell polarization, and induction of cell motility [41]. Also, the AKT activation in squamous cancer cells was shown to undergo EMT and downregulate Ecadherin [41]. In addition, some studies have reported that AKT and Twist were involved in a positive feedback loop, which resulted in a series of events that enthaced their pro-EMT function [42, 43]. Furthermore, Salt *et al.* have suggested that mesenchymal cell proliferation can be reversed by the addition of a PI3K inhibitor [44]. Given that PI3K/AKT signialing pathway is essential for EMT-mediated metastasis, HS-173 treatment seems to be an effective therapy of pancreatic cancer. Additionally, HS-173 downregulated the expression of ZEB1 which is known as an Ecadherin repressor. ZEB1 is one of the well-known transcription factors on EMT and metastasis, emerging resistance to therapy and poor prognosis in breast, pancreatic, and lung cancer [45]. According to Lehmann *et al.*, ZEB1 was highly expressed in Panc-1 pancreatic cancer cells, and mentioned it as an aggressive cancer cell line [46]. In agreement with the above study, TGF-β increased the expression of ZEB1 in Panc-1 cells, which was clearly inhibited by HS-173 treatment. From this result, we assumed that HS-173 had a strong correlation between the downregulation of TGF-β-induced EMT and the inhibition of ZEB1 in pancreatic cancer cells. Besides, HS-173 showed a significant effect on the phosphorylation of Smad2/3, which is consistent with the results from the study by Guanyu *et al.*, showing that BEZ-235, a PI3K/mTOR inhibitor, inhibited expression of Smad2/3 and Akt increased by TGF-β in ovarian cancer cells [47].

Most importantly, the effect of HS-173 on EMT *in vitro* was also observed *in vivo* in three mouse pancreatic cancer models including the xenograft, orthotopic, and metastasis models. Our study revealed that HS-173 significantly inhibited the primary tumor growth and metastasis without body weight loss. Since pancreatic tumors show a high tendency to metastasize to distant organs such as liver, lung, and lymph node, we identified the metastasis region and nodules spreading to liver, lung and lymph node, as parameters of pancreatic tumor metastasis. Compared with control tumors, HS-173 treated group (10 and 30 mg/kg) significantly reduced metastasis to liver, lung and lymph node. Besides, the potential of HS-173 on EMT inhibition was confirmed by *in vivo* results, showing that the expression of Ecadherin was significantly increased by HS-173, whereas a mesenchymal marker Vimentin was decreased. These results are in accordance with induction of apoptosis due to increased expression of cleaved caspase-3 and DNA fragmentation, as observed in TUNEL staining, in parallel with a decrease in PCNA immunostaining for cell proliferation in tumor tissues. Consequently, we considered that targeting of PI3K/AKT signaling pathways by HS-173 may enhance the antitumor activity via EMT inhibition in pancreatic cancer.

Until now, there has been a variety of potential pharmacological approaches developed against the EMT process to prevent or target cancer metastasis. These approaches include the inhibition of EMT induction and targeting the invasive mesenchymal cells. In this study, we found that HS-173 could target invasive mesenchymal cells and inhibit TGF-β induced EMT, migration, and invasion of pancreatic cancer cells. Also, upon TGF-β stimulation, HS-173 blocked the ZEB1 transcription. In addition, our results revealed that HS-173 inhibited not only PI3K/AKT signaling but also Smad2/3 signaling pathway (Figure 7F). Moreover, in the xenograft, orthotopic, and metastatic mouse models, HS-173 showed a significant inhibitory effect on tumor growth and EMT-mediated metastasis in pancreatic cancer. Therefore, we suggest that HS-173 might be a potential target for therapeutic strategy against advanced pancreatic cancer with metastasis.

## MATERIALS AND METHODS

### Chemicals and antibodies

Recombinant human TGF-β1 were purchased from R&D system (catalog# 240-B) and dissolved in deionized distilled water and aliquoted for experiments. All antibodies were purchased from Cell Signaling Technology (MA, US), Abcam (Cambridge, UK) and Santa Cruz (CA, US); Ecadherin (#3195), Vimentin (#V2258), Ncadherin (#98952), ZEB1 (#NBP1-05987), Snail (#180714), Cytokeratin18 (#ab668), Cytokeratin19 (#ab52625), p-Smad2 (#3101), p-Smad3 (#9520), Smad2/3 (#3102), p-AKT (#3102), AKT (#3102), p-mTOR (#3102), mTOR (# 2972), and β-actin (#G043).

### Preparation of HS-173

Ethyl 6-(5-(phenylsulfonamido) pyridin-3-yl)imidazo[1,2-a]pyridine -3-carboxylate (HS-173) is a novel PI3Kα (class I) inhibitor. This imidazopyridine derivative was synthesized as described in our previous study [27]. For all *in vitro* studies, HS-173 was dissolved in dimethylsulfoxide (DMSO) at 20 mM stock concentration and diluted freshly to working concentrations, whereas in the case of *in vivo* experiments, the desired doses were prepared dissolving in DPBS: PEG400:DMSO (4:5:1).

### Cell culture

Human pancreatic cancer cells (Panc-1, Miapaca-2, and Aspc-1) were purchased from American Type Culture Collection (Manassas, VA). Panc-1 and Miapaca-2 cells were cultured in Dulbecco's modified Eagle's medium (DMEM) supplemented with 10% heat-inactivated fetal bovine serum (FBS) and 1% penicillin/streptomycin. Aspc-1 cells were cultured in Roswell Park Memorial Institute 1640 (RPMI-1640) media supplemented with 10% FBS and 1% penicillin/streptomycin. FBS and all other reagents used for cell culture were purchased from Invitrogen (Carlsbad, CA, USA). The cultures were maintained at 37°C in an incubator with a controlled humidified atmosphere composed of 95% air and 5% CO_2_.

### Measurement of cell proliferation

Cell viability was performed using an MTT assay. In brief, cells were seeded at a density of 5000~7000 cells/well in a 96-well plates following to 24 h incubation. On the following day the media were removed and the cells were treated with either vehicle as a negative control or various concentrations of HS-173 (0.5–10 μM) following an incubation for 24, 48, or 72 h. After incubation of respective time 10% of an MTT solution (2 mg/mL) was added to each well and the cells were incubated for another 4 h at 37°C. The formazan crystals that formed were dissolved in DMSO (100 or 300 μL/well) with constant shaking for 5 min. The absorbance of the plate was then read with a microplate reader at 540 nm [15]. Three replicate wells were evaluated for each analysis.

### Clonogenic assay

Briefly, depending on pancreatic cancer cell lines, the cells (200-250 cells) were seeded in 6-well plates and after overnight incubation the cells were treated with different concentrations of HS-173. After 24 h treatment, the media were changed and were left the plates for 7-10 days depending on the cell lines. Colonies were fixed and stained with crystal violet. The plates were photographed and the colonies were counted using ImageJ. Each assay was performed in triplicate and only the colonies containing at least 50 cells were analyzed.

### Wound migration assay

A monolayer of cells were plated on 60-mm culture dishes, and at 90% confluency a linear wound was made (~ 4 mm wide) using a 200 μL pipette tip. The detached cells were washed away by DPBS. Then, the monolayer was further treated by TGF-β (10 ng/mL) either alone or with HS-173 (0.5–10 μM). The cells were allowed to migrate for 16 h, washed with serum-free medium and fixed in absolute methanol. The migrated cells were observed under a phase-contrast microscope and photographed at 200× magnification.

### Transwell invasion assay

Cell invasions were analyzed in transwell permeable support systems (24-well plate cell culture inserts with 8.0 μm porous transparent PET membrane, Corning# 353097, NY, USA). The inserts were coated with 10% matrigel for 30 min before cell seeding. The cells were starved for 12 h and seeded at 1 × 10^5^ cells/insert in serum-free medium treated with TGF-β (10 ng/mL) only or in combination with HS-173 (0.5–10 μM). The outside of the insert membranes were filled with DMEM medium supplemented with 10% FBS. After 48 h incubation, the cells were wiped away from the insert tops and the cells that invaded through the polycarbonate basement membrane were stained with crystal violet. The invaded cells were photographed at 100x magnification and counted as numbers per field.

### Three-dimensional (3D) multicellular spheroid cultures

Three-dimensional multicellular spheroid cultures were generated using a modified hanging droplet method [16]. The pancreatic cancer cells were suspended in DMEM/10% FBS at a concentration of 50,000 cells/mL. Then, 20 μL of cell suspension was pipetted onto the underside of a sterile 10-cm tissue-culture plate lid. Each lid holds approximately 50 droplets. After loading the droplets, the lid was placed carefully onto a culture plate containing 5 mL of DPBS and incubated for 48 and 96 h in Panc-1 and Aspc-1 cells respectively to form cellular aggregation and then spheroid. The freshly formed spheroids were then transferred into the ultra low attachment plate (Corning Inc., Corning, NY, USA; Cat#3473) containing DMEM/2% FBS. Each well contained about 50 spheroids. After transfer, the spheroids were treated with vehicle or with 10 ng/mL TGF-β alone or TGF-β at various concentration of HS-173 (0.5-10 μM) and incubated for 72 h. Then, the spheroids were harvested and used for Western blot and immunofluorescence microscopic analyses.

### Sphere formation assay

The cells were plated in 24-well ultra low attachment plates (Corning) at a density of 5,000 cells/well in DMEM (supplemented with 1% N2, 2% B27, 20 ng/mL hFGF, 100 ng/mL hEGF and 1% penicillin/streptomycin). After overnight incubation the cells were treated by vehicle or with 10 ng/mL TGF-β alone or TGF-β at various concentrations of HS-173 (0.5-10 μM) for 10 days. Twice a week, fresh media supplemented with all factors were added to cells. After 10 days of incubation, the cells were observed under the phase contrast microscope and photographed.

### Western blotting

The cells were washed with DPBS before being lysed in a lysis buffer containing protease and phosphatase inhibitors. Equal amounts of protein were separated using 8, 10 or 12% sodium dodecyl sulfate (SDS)–polyacrylamide gel electrophoresis and transferred onto polyvinylidene fluoride (PVDF) membranes. Protein transfer was confirmed using a Ponceau S staining solution (Sigma Aldrich, Ohio, USA). The blots were then immunostained with the appropriate primary antibodies (1:1000) followed by appropriate secondary antibodies (1:5000) conjugated to horseradish peroxidase. The primary antibodies specific to the interested proteins were used and detected manually using X-Ray film by enhanced chemiluminescence reagent (Amersham Biosciences, Piscataway, NJ).

### Immunofluorescence microscopy

The cells were plated on 18-mm cover glasses in DMEM at a density of 1 × 10^5^ cells/well and incubated for 24 h. Next day the cells were treated with vehicle or 10 ng/mL TGF-β alone or TGF-β at various concentrations of HS-173 (0.5-10 μM) and incubated for 24 h. The cells were then washed with DPBS and fixed in an acetic acid:ethanol (2:1) solution for 5 min. Nonspecific binding was blocked with 5% goat and horse serum/PBS for 1 h at room temperature, and the cells were then incubated overnight with the primary antibodies (1:50) in a humidified chamber. After washing twice in PBS, the cells were incubated with fluorescence-labeled secondary antibody (1:100) for 1 h at room temperature in the dark. The nuclei were stained with 4, 6-diamidino-2-phenylindole (DAPI) in the dark for 30 min at room temperature. The slides were washed twice with PBS, covered with DABCO (Sigma-Aldrich) and examined with confocal laser scanning microscopy (Olympus, Tokyo, Japan) at 488 and 568 nm.

### Xenograft models

Male BALB/c mice (4 week old, weighing 18-20 g) were obtained from Orient Bio. Animal Inc. (Seoul, Republic of Korea). Animal care and all animal related experimental procedures were conducted in accordance with the approval and guidelines of the INHA Institutional Animal Care and Use Committee (INHA IACUC) of the Medical School of Inha University (approval ID: INHA 150629-367-2). The animals were fed with standard rat chow and tap water *ad libitum*, and were maintained with a 12 h dark/light cycle at 21°C [3]. After one week of adaptation period, Panc-1 cells (5 × 10^6^ cells/mice) were inoculated in the right flank of the mouse. After reaching an average tumor volume of 50 mm^3^, mice were randomly divided into two groups with five mice in each group; the control group was treated with vehicle and the experimental group was treated with HS-173 (10 mg/kg) intraperitoneally thrice a week for 26 days. The body weight and tumor size were measured thrice a week using the formula, 0.5 × length × width^2^. At the end of the experiment, mice were sacrificed and primary tumor was harvested. Tumors were weighed, photographed, and divided into two parts for Western blot and IHC analysis. For IHC analysis, tissues were immediately fixed in 4% PFA for overnight and for Western blotting analysis, tissues were snap-frozen in liquid nitrogen.

### Orthotopic models

After one week of adaptation period, Panc-1 cells (8 × 10^6^ cells/mice) were orthotopically implanted in the pancreas of nude mice. A total of six mice were anesthetized by Zoletil : Rompun (9:1) solution and a small left abdominal flank incision was made. About 8 × 10^6^ cells suspended in 200 μL DPBS were injected into the subcapsular region of the pancreas using 31G insulin syringe. The peritoneum and skin incisions were closed sequentially with 9 mm wound autoclips. After 10 days, mice were randomly separated into two groups with 3 mice in each group. Mice in the experimental group received 10 mg/kg HS-173 for 6 weeks (3 times/week, i.p.), whereas the control mice received vehicle only. The dose of HS-173 was selected based on previous reports [13, 14]. The body weight was measured thrice a week. At the end of the experiment, mice were sacrificed and primary pancreatic tumor was excised from each mouse. Tumors were weighed, photographed and immediately fixed in 4% PFA overnight for further immunohistochemistry.

### Cancer metastasis model

Miapaca-2 cells (1 × 10^7^) suspended in 50 μL DPBS were implanted into the spleen of the pancreas using 31G insulin syringe. After 30 days, mice were randomly separated into three groups with 7 mice in each group; the control group was treated with vehicle only and the other two groups were treated with HS-173 (10 or 30 mg/kg) intraperitoneally thrice a week for 25 days. The body weight was measured thrice a week. At the end of the experiment (55 days), mice were sacrificed and primary tumors, liver, lung and lymph nodes were excised from each mouse. Tumors were weighed, photographed and immediately fixed in 4% PFA overnight for further immunohistochemical analysis.

### Biochemical analysis

Blood samples were collected from the postcaval vein and followed by the centrifugation at 570×g for 10 min to obtain serum. Serums were stored at -70°C before analysis. The levels of AST and ALT were determined following Reitman and Frankel's method [48] using assay kits supplied by BioVision (Milpitas, CA). The level of BUN was measured by a microplate-based colorimetric enzyme assay kit (BioVision).

### Immunohistochemistry

Immunohistochemical staining was performed using formalin-fixed and deparaffinized tissue sections as previously described [15]. Briefly, the tumor tissue sections were blocked with normal goat or horse serum (Vector Laboratories) for 1 h and incubated at 4°C overnight in 1:50 dilutions of primary antibodies against Ecadherin, Vimentin, ZEB1, TUNEL, PCNA and p-Smad2. The sections were then incubated with biotinylated secondary antibodies (1:100) for 1 h. The sections were visualized by an avidin-biotin peroxidase complex solution using an ABC kit (Vector Laboratories), which were then washed in PBS and developed with a diaminobenzidine tetrahydrochloride (DAB) substrate for 15 min, and then counterstained with hematoxylin. At least 3 randomly selected fields for each section were examined at 200x magnification and analyzed.

### TUNEL staining

The terminal deoxynucleotidyl transferase-mediated nick end labeling (TUNEL) staining were performed using the formalin-fixed and deparaffinized tissue sections as previously described [17]. In brief, the tumor tissue sections were deparrafinized and TUNEL was subsequently performed using a TUNEL kit ApopTag^®^ Peroxidase In Situ Apoptosis Detection Kit#S7100 (Merck Millipore, Temecula, CA, USA) in accordance with the manufacturer's instructions.

### Statistical analysis

Data are expressed as the mean ± SD, and analyzed with an ANOVA and unpaired Student's t-test. A *P*-value of 0.05 or less was considered statistically significant. Comparisons of results were performed using a Student's t-test.
